# Fine-Scale Linkage Mapping Reveals a Small Set of Candidate Genes Influencing Honey Bee Grooming Behavior in Response to Varroa Mites

**DOI:** 10.1371/journal.pone.0047269

**Published:** 2012-11-02

**Authors:** Miguel E. Arechavaleta-Velasco, Karla Alcala-Escamilla, Carlos Robles-Rios, Jennifer M. Tsuruda, Greg J. Hunt

**Affiliations:** 1 Fisiología y Mejoramiento Animal, Instituto Nacional de Investigaciones Forestales, Agricolas y Pecuarias, Ajuchitlan, Queretaro, Mexico; 2 Valles Centrales, Instituto Nacional de Investigaciones Forestales, Agricolas y Pecuarias, Etla, Oaxaca, Mexico; 3 Department of Entomology, Purdue University, West Lafayette, Indiana, United States of America; Arizona State University, United States of America

## Abstract

Populations of honey bees in North America have been experiencing high annual colony mortality for 15–20 years. Many apicultural researchers believe that introduced parasites called Varroa mites (*V. destructor*) are the most important factor in colony deaths. One important resistance mechanism that limits mite population growth in colonies is the ability of some lines of honey bees to groom mites from their bodies. To search for genes influencing this trait, we used an Illumina Bead Station genotyping array to determine the genotypes of several hundred worker bees at over a thousand single-nucleotide polymorphisms in a family that was apparently segregating for alleles influencing this behavior. Linkage analyses provided a genetic map with 1,313 markers anchored to genome sequence. Genotypes were analyzed for association with grooming behavior, measured as the time that individual bees took to initiate grooming after mites were placed on their thoraces. Quantitative-trait-locus interval mapping identified a single chromosomal region that was significant at the chromosome-wide level (p<0.05) on chromosome 5 with a LOD score of 2.72. The 95% confidence interval for quantitative trait locus location contained only 27 genes (honey bee official gene annotation set 2) including *Atlastin*, *Ataxin* and *Neurexin-1 (AmNrx1)*, which have potential neurodevelopmental and behavioral effects. *Atlastin* and *Ataxin* homologs are associated with neurological diseases in humans. *AmNrx1* codes for a presynaptic protein with many alternatively spliced isoforms. *Neurexin-1* influences the growth, maintenance and maturation of synapses in the brain, as well as the type of receptors most prominent within synapses. *Neurexin-1* has also been associated with autism spectrum disorder and schizophrenia in humans, and self-grooming behavior in mice.

## Introduction

Ectoparasitic Varroa mites are considered by many to be the greatest threat to honey bee health worldwide. Increased annual mortality rates of North American colonies began about the time that tracheal mites (*Acarapis woodii*) and Varroa mites (*V. destructor*) first became established in the U.S. (about 1990) [1]. *Varroa destructor* switched hosts from Asian honey bees (*Apis cerana*) to the species commonly used for honey production and pollination worldwide (*A. mellifera*), about 60 years ago. Female Varroa mites lay their eggs within sealed brood cells in which bee larvae go through metamorphosis prior to emerging as adults. The mite progeny must mature and mate within the brood cell before the bee emerges for the mite to successfully reproduce. Virtually all honey bee colonies are infested with Varroa and unless steps are taken to reduce mite levels, colonies usually die within six months to two years, exhibiting dwindling populations and symptoms of viral and brood diseases [2].

Most colonies of bees in North America now show sufficient resistance to endoparasitic tracheal mites such that no treatment is needed to control them [3] but Varroa mite infestation has continued to be associated with high winter mortality in North America [1], [4]. The presence of *V. destructor* and the virus most commonly associated with mite infestation remain strong predictors of winter mortality in Europe [5]. Approximately 30% of bee colonies have been lost annually in the US in recent years [6]–[9]. Nationwide surveys of US bee losses were not published prior to 2006, but regional surveys taken after mites became established sometimes showed winter colony losses over 50%. Apiary losses were much reduced if beekeepers controlled Varroa mites [10]–[11]. As of 2009, high annual colony mortality has not been reported in Mexico [12]. However, Varroa is the health problem that has the greatest negative effect on honey production in Mexico and colony honey yields are significantly affected by mites [13]–[14]. Recently, surveys in North America and Europe also show a consensus that *V. destructor* infestation is still the most significant cause of annual colony mortality among all measured risk factors [15]–[20].

European races of *A. mellifera* are commonly used in beekeeping in most of the world, but an African race (*A. mellifera scutellata*) was introduced to Brazil in 1956. This race hybridized with European races, to form the Africanized honey bee, which has spread northward and has colonized all of Mexico and part of the US. The Africanized honey bee appears to have retained most of the original traits of the African race [21], including a somewhat higher degree of tolerance to Varroa than European races. But Africanized colonies still succumb to Varroa infestation [22]–[24]. A study conducted in Mexico identified grooming behavior as the most important factor that reduced mite population growth in a genetically diverse set of colonies. Colonies that had the lowest mite population growth during an eight-month period exhibited higher grooming behavior, had higher proportions of chewed mites falling from bees in the colonies, and reduced infestation levels of adult bees [25].

Mite-grooming behavior also varies between stocks of European honey bees in North America and can reduce infestation levels on adult bees [26]–[28]. The proportion of chewed mites falling from bees was found to correlate with removal rates in laboratory grooming assays [28], which corroborates earlier observations from hives in Europe [29]. Grooming behavior is an important resistance trait in Asian honey bees, the original host of *Varroa,* and is more strongly expressed in this species [30]–[31]. Recently, comparisons were made between four pairs of relatively mite-tolerant or susceptible honey bee stocks. The bees tested came from diverse geographic sources (Africanized bees tested in Mexico, Russian and European races tested in Canada). All mite-tolerant stocks showed comparitively higher proportions of chewed mites falling from colonies and increased intensity of individual grooming actions in laboratory assays, which underscores the importance of this trait [32].

Another honey bee mite-resistance mechanism was identified that involves bees uncapping infested brood cells, resulting in removal of pupae and mites from the cell or in the disruption of mite reproduction [33]–[35]. The latter trait has been called Varroa-sensitive hygiene, or VSH. Research has shown that Africanized honey bees exhibit a higher average levels of both grooming behavior and VSH compared to European races but it is unclear which trait is the most important for the Africanized honey bee’s increased tolerance to mites [22]–[24], [36].

Our objective was to use a QTL mapping approach to identifying candidate genes for honey bee mite-grooming behavior. In this report we describe fine-scale mapping of one putative QTL influencing mite-grooming behavior and the identification of a small set of candidate genes within the QTL region. Our approach was to use a single backcross family of worker bees derived from crosses between two colonies selected for high or low levels of grooming behavior. The time that individual beestook to exhibit grooming behavior after to contact with a single mite was quantified and these behavioral phenotypes were analyzed for association with alleles for about 1,300 intragenic single-nucleotide polymorphisms (SNPs). The results presented here and in a companion paper [37] are the first reports to identify candidate genes influencing behaviors that have been shown to be the key traits for suppressing mite population growth in screens of diverse populations of bees. These studies also have produced the most detailed and accurate linkage maps of the honey bee genome based on SNPs analyzed on genotyping arrays.

## Results and Discussion

**Figure 1:**
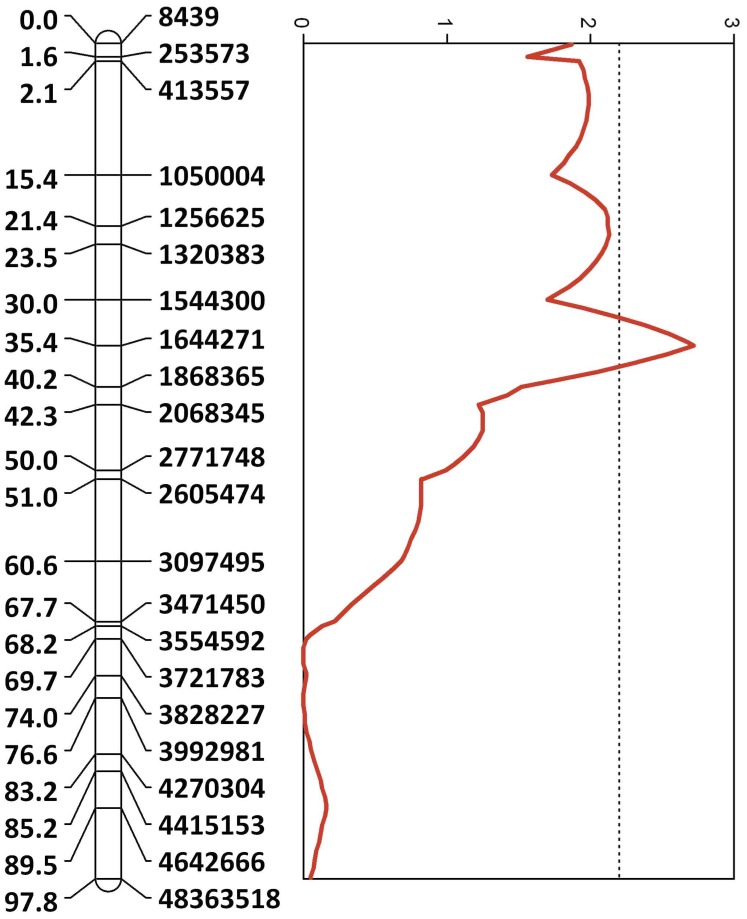
Map of QTL location for groom-1 on chromosome 5. The physical location in base pairs of SNP probes in the honey bee genome assembly (Amel 4.0) is indicated to the right of the bar. The large number associated with the last SNP marker refers to its location in a contig that was not assigned to a chromosome prior to this study. Numbers to the left of the bar are distances in centimorgans. The red line indicates the LOD score for the likelihood that a QTL influencing grooming behavior is linked. The dotted line indicates the chromosome-wide empirical significance threshold of 0.05 as determined by 1000 permutations of phenotype data. Probe sequences matching the chromosome positions are available in **Table S1**.

One putative QTL that we will refer to as groom-1 was identified with a LOD score of 2.72, covering about 2.0 Mb of chromosome 5 (Figure 1). On average, individuals that were homozygous for the high-grooming allele reacted to the presence of a mite faster than heterozygotes (18 versus 30 sec). Permutation tests indicated that this QTL exceeded the chromosome-wide significance level (p<0.05) but was not significant in a genome-wide test. Our results are relatively fine-scale in resolution; there were only 27 candidate genes that had been annotated in the official gene set 2 located in the LOD-1.5 support interval for this QTL, although future bioinformatic and EST analyses will probably identify additional genes and non-coding RNA transcripts in this region. The reason the candidate gene set is so small can be ascribed partly to the fact that this is not a gene-rich region, but is also the result of high marker saturation and the high recombination rate in the bee. The average recombination rate in the QTL region, 42 Kb/cM, was similar to the overall average for the bee, which has the highest reported rate for any metazoan species [38]–[39]. Two predicted genes could not be assigned any function (GB14792 and GB14110, Table 1). Two other genes, GB10440 and GB10743, are predicted to code for short proteins consisting of 80 and 242 amino acids, respectively, with high homology to proteins annotated in the bumble bee as *ataxin-10* like. The presence of simple repeats within an intron of this gene in humans is associated with spinocerebellar ataxia type 10, characterized by atrophy of the cerebellum [40]–[41]. Two additional genes show homology to *atlastin*. The first is a putative gene designated GB15435, and annotated as containing only 81 amino acids. The other, GB14853, is the honey bee ortholog of *atlastin-1,* a membrane-bound large GTPase of the dynamin superfamily. Mutations in the human *atlastin-1* are the most common risk factor for early onset of hereditary spastic paraplegia (HSP). *Atlastin-1* protein is embedded in the endoplasmic reticulum (ER) and is critical for development of branched ER. In humans, defects in the ER are thought to be largely responsible for the etiology of HSP, which primarily affects long motor axons in humans that may extend up to several meters [42]. *Atlastin-1* is one of several genes within the confidence interval that are important for function of the ER, microtubule function and protein trafficking (Table 1).

**Table 1:** Candidate genes for honey bee mite-grooming behavior and their putative functions.

Gene name	Drosophila homolog	Predictions and proteindomains from blastp searches	Putative function
GB12154		Wnt-7b-like	Embryogenesis, morphogenic signaling
GB10440	CG4975	Ataxin-10-like	Neuron survival, differentiation, neurogenesis
GB10743	CG4975	Ataxin-10-like	
GB16526	CG1093	Proteasome subunit alpha type-5 Ntn hydrolase	Fatty & amino acids metabolism
GB12705	CG30301	zinc finger protein 512B	Transcriptional regulation
GB15435		atlastin-like	Endoplasmic reticulum fusion, regulation of synaptic growth at neuromuscular junction
GB11239		Wnt-7b-like	Wnt signalling pathway
GB17462		regulator of microtubule dynamics protein 1-like	Retinol dehydrase 13-like
GB14853	CG6668	*Atlastin-1*	See GB15435
GB14672		ELM2 domain – unknown function SANT – DNA-binding domains	Transcriptional-regulating factor 1
GB18506	CG4406	Peptidase C13 superfamily	Putative GPI transamidase mediates GPI anchoring in the endoplasmic reticulum
GB13244		KAZAL_FS	Follistatin-like
GB10034	CG8079	G patch & FHA domains	angiogenic factor-like
GB17256	CG8014 *Rme8*	dnaJ homolog subfamily C member 13	*Drosophila* ortholog involved in receptor mediated endocytosis
GB20007		zinc finger protein 62-like THAP superfamily	THAP domain is a putative DNA-binding domain
GB17376		NADB Rossman superfamily FAR-C superfamily	fatty-acyl-CoA reductase 1-like
GB10140	CG12173	Phosphopentain phosphatase domain	Enolase-phosphatase E1; Methionine metabolism
GB19109		zinc finger protein 484-like	C2H2 domain, protein-protein interaction
GB16719	CG14181	Vesicle transport- USE1	Vesicle docking in the ER
GB17810	CG6176 *Grip75*	gamma tubulin complex component 4	Orientation of microtubules
GB18337	CG2774	sorting nexin 2-like	Intracellular protein trafficking
GB16547	CG18212	kinectin-like	Protein trafficking in the ER
GB11559	CG1972	Lactamase B superfamily RMMBL superfamily	RNA processing in translation and ribosome biogenesis (exonuclease)
GB18754	CG7050	Neurexin 1 EGF_CA and LNS superfamily domains	*AmNrx1*-Presynaptic protein involved in initiation, maintenance and function of synapses.
GB19804		Secapin preproprotein	Conserved venom peptide

The groom-1 QTL region also contains the sequence for the honey bee ortholog for *neurexin-1*, *AmNrx1* (GB18754). As in other animals, *AmNrx1* is highly expressed in the central nervous system and at a much lower level in other tissues. It is a large gene (∼400 Kb, 28 exons) that shows extensive alternative splicing. Twelve splice variants that differ in functional domains have been identified [43]. The two longest honey bee splice variants resemble human alpha-isoforms and one of the shortest forms resembles the beta isoform [44]. These two functional variants are generated through the use of alternative 3′ exons in the bee. The human gene generates these similar classes of isoforms by use of alternative promoters, suggesting convergent evolution. The intracellular domains of these two isoform classes are similar and in humans have been shown to interact with a number of proteins such as synaptogamin via PDZ domains, which can influence neurotransmitter release. The extracellular portion of the protein interacts with neuroligins embedded in the post-synaptic membrane and other synaptic proteins [45]–[49]. Nine isoforms in the bee lack the transmembrane domain. These apparently soluble isoforms may regulate neurexin functions and have been found in humans, although their functions apparently have not been determined [50]. Expression of *AmNrx1* and neuroligin genes are concentrated in the mushroom body of the brain, which is the center of higher-order processing and learning in the bee [51]–[52]. *AmNrx1* expression is also influenced by sensory experience, suggesting that it may play a role in the development of increased synaptic connections and behavioral plasticity [53].

The association of extracellular portions of neurexin and neuroligin proteins in nerve synapses serve important functions. These interactions form cellular adhesion complexes that regulate synapse formation, maintenance and maturation [45]–[48]. In humans and bees, the splice variants of *neurexin-1* differ in the number of LNS motifs that interact with neuroligins in the synaptic gap. The longest alpha isoforms have three repeats, each consisting of an EGF (epidermal growth factor-like) sequence flanked by two LNS (laminin-neurexin-sex hormone binding globulin) motifs. The various isoforms of human *neurexin-1* show specific differences in binding affinities for proteins in the synaptic cleft that regulate the function of the synapse [48]. Mutations of *neurexin-1-alpha* have been linked to autism spectrum disorders and schizophrenia in humans [54] and to synapse formation and associative learning in both *Drosophila* and the sea slug, *Aplysia* [55]–[56]. Knockout (KO) mice for *neurexin-1-alpha* were developed as a potential model system for autism spectrum disorder. Surprisingly, homozygous KO mice show greatly increased self-grooming activity compared to littermate controls, along with decreased nest building behavior and better performance in motor-learning tasks [57]. The grooming-behavior and motor-learning results suggest an increased sensitivity to tactile stimuli in the KO mice.

The possibility that neurexin-1 might influence grooming behavior in both honey bees and mammals is surprising, but of course discussion of candidate genes is speculative. This study identified just one putative QTL for honey bee mite-grooming behavior that was significant at the chromosome-wide level (LOD threshold = 2.28 for p<0.05) but not at the genome-wide level (LOD threshold = 3.49). It is necessary to confirm the effects of this QTL in a different population of bees. QTLs influencing behavioral traits usually have low LOD scores because these are traits influenced by large environmental effects and complex genetic architectures. In previous honey bee studies, only two QTL influencing the age of onset of foraging and one QTL influencing colony-level stinging response have met the threshold for genome-wide significance [58]–[59]. However, a number of honey bee behavioral QTL with low LOD scores have been confirmed in independent tests [60]. A recent study identified several QTL for a physiological mechanism of resistance in honey bees to Varroa mites but unfortunatelly these QTL only reached significance at the level of chromosomal region and the study could not narrow the list of candidate genes to a manageable number [61].

More effort is needed to improve the effectiveness of grooming assays. Our assay measured the time required for a bee to respond to the stimulus caused by the presence of a mite, which correlated with the proportion of mutilated mites falling from the test colonies (M. Arechavaleta-Velasco and K. Alcala-Escamilla, unpublished data). However, there was variation in how much the mites moved when on a bee. Perhaps an assay that measured the intensity of grooming actions in response to a biotic or abiotic stimulus might be better [32]. Differences in behavioral assays and/or seasonality in expression of the behavior are probably responsible for the discrepancy between heritability estimates and response to selection for mite-grooming traits [62]–[66]. It may also be useful to study correlated traits that may be influenced by the same causal gene(s). For example, one could test for effects on associative learning through training regimes that use the well-developed proboscis extension reflex assays. If the effect of the QTL on grooming behavior is confirmed, the identification of candidate genes that influence neural function in this region suggests promising directions for future research, such as correlating alternative splice variants or expression levels of *AmNrx1* with honey bee mite-grooming behavior. RNAi knockouts of specific isoforms may also be possible if they can be targeted to the brain. If *AmNrx1* does influence mite-grooming behavior, the honey bee would be a valuable comparative model to help understand behavioral and neurological influences of a protein that is highly conserved and involved in human neurological disorders. This research could also have an impact on breeding for resistance to mites.

Identification of a specific gene variant that has a major impact on behavioral resistance to Varroa mites would provide a means to more rapidly select for resistant strains of bees through a simple genotyping assay. It is important to actually identify the causal gene, or even the causal sequence variant, because the bee’s high recombination rate would likely result in many historical crossover events between a causal sequence and an intergenic DNA marker, reducing the linkage disequilibrium needed to predict the presence of favorable alleles. Marker-assisted breeding programs could be used to select colonies for high grooming behavior so that bees would be able to reduce their mite infestations to levels that would not affect honey production or compromise colony survival.

## Materials and Methods

### Ethics Statement

No permits were required to conduct the field research or genotyping analyses. The crosses and field research were conducted in Mexico under the supervision of researchers of the Instituto Nacional de Investigaciones Forestales, Agrícolas y Pecuarias, INIFAP Mexican agricultural research service). Genotyping was performed in the Purdue core genomics facility in accordance with university and federal biosafety regulations.

### Behavioral Assay

Honey bee grooming behavior was measured in this study using the laboratory assay described by Espinoza *et al.* [66], with few modifications. The behavioral assay was conducted in an “observation frame” that was built using standard flat plastic hive frame foundation (45.0×16.0×1.5 cm) that has a pattern of hexagonal ridges and is coated with beeswax. This foundation served as the stage for observing bees. The foundation was inserted into a wooden hive frame of the appropriate size. The wood frame was covered on one of the ends with a piece of wire mesh of 16.0×10.0 cm for ventilation, the rest of the frame was covered with a transparent clear plastic sheet 1 mm thick. The wood frame was used to create a sealed area of 43.0×14.0×3.0 cm where bees could be confined an observed. In the center part of this area a circular area of 7.0 cm in diameter was built using wire mesh to create an inner arena inside the observation area, that excludes the passage of bees, but not the passage of air and to allow honeybee antennation and trophallaxis (touching of antennae and food sharing). A 2.5×2.5 cm window was cut in the plastic sheet above the circular area and a sliding piece of the plastic sheet was used as a cover to permit opening and closing of the window.

Adult Varroa mites were collected from a highly infested colony, by shaking adult bees from the hive frames into a 10 l plastic container, that had a 4.0 mm wire mesh screen 10 cm above its bottom. The container was closed with its plastic lid and CO_2_ was applied through a small hole in the cover and then the container was sealed for 3 min to anaesthetize the bees and to allow the mites to fall off from the workers. The mites were collected from the bottom of the container and placed in a petri dish and kept at room temperature in the laboratory. Approximately 300 worker bees from the colony to be tested were placed in the observation frame using a soft brush and four bees were placed in the inner circular arena. The observation frame was then sealed and taken to the laboratory. Sugar syrup (50% v/v) and water was fed to the bees through the wire mesh of the observation frame. An additional 40 honey bee workers were collected from the test colony, and each bee was introduced into a 10 ml Falcon™ tube and taken to the laboratory where they were kept at 8°C for a few minutes until their mobility was reduced to facilitate the manipulation of the bee.

The bee to be tested was transferred to the circular observation arena through the window in the plastic sheet with the help of a plastic tweezers. When the bee appeared to have fully recovered from the effect of the low temperature, a Varroa mite taken from the Petri dish was placed on her thorax through the window using a fine brush. The bee was observed by two people and the time that she took to respond by performing grooming behavior after the mite was placed over her body was recorded. Grooming behavior was defined as swiping motions in the direction of the mite with the front two pairs of legs. Colony phenotype was estimated by the mean reaction time of 40 workers.

### Mapping Population

Two honey bee colonies were used as parental sources, one classified as a high groomer and one classified as a low groomer based on bi-directional selection in a population (n = 60) of honey bee colonies using the behavioral assay on 40 workers per hive. A queen was reared from each of the parental colonies and was artificially inseminated with the semen of three of her brothers. This inbreeding step was performed to ensure more genetically uniform F1 queens. A queen was reared from the high-grooming inbred colony and was artificially inseminated with the semen of a single haploid drone from the low-grooming inbred colony. From this queen, twenty F1 queens were reared and divided into two groups. Ten F1 queens were single-drone artificially inseminated with drones from the high grooming inbred colony and ten queens were single-drone inseminated with drones from the low-grooming inbred colony, to produce two types of colonies composed of backcross workers.

Each queen was introduced into a small colony made with three frames of brood, two frames of honey and approximately 1.5 kg of bees. The colonies were kept in single deep Jumbo type hives in the same apiary. All colonies were managed in the same way for a period of 60 days prior to the beginning of the experiments to allow time for workers in the colony to be replaced by daughters of the inseminated queens.

The grooming behavior of the 20 backcross colonies was tested using the behavioral assay, and the colony with the greatest range in grooming behavior phenotypes was selected for QTL mapping (a backcross to the high line). Worker bees (n = 400) from this colony were tested with the assay and the time that the each bee took to react by performing grooming behavior after a mite was placed on her thorax was recorded. At the end of each test the bee was collected in a 1.5 ml tube with ethanol and placed at −20°C. From the 400 bees that were tested, the 98 from the top of the phenotypic distribution and the 98 from the bottom of the distribution were selected for genotyping. Genomic DNA was extracted from each bee, DNA extraction involved grinding the bees in lysis solution (1% CTAB, 50 mM Tris pH 8.0, 10 mM EDTA, 1.1 M NaCl), followed by phenol-chloroform extraction and ethanol precipitation of the DNA [67]. The DNA of each individual bee was quantified and diluted in double distilled water to a final concentration of 50 ng/µl and kept at −80°C.

### Genotyping and Linkage Analyses

Prior to genotyping the mapping population, the F1 queen that was the mother of the backcross colony was subjected to genomic sequencing on an ABI SOLiD platform. Heterozygous SNPs were identified using DiBayes software and these were compared to heterozygous SNPs in an unrelated F1 queen that was the mother of another mapping population (for a different QTL study) so that it was possible to design a set of probes that would be informative in both populations. Genotyping was performed using 250 ng of DNA of the 196 selected worker bees using the Illumina GoldenGate Assay, which usually achieves accuracies of ∼99.7 to 99.9% [68]. Briefly, DNA was fragmented and activated for binding to paramagnetic particles, then hybridized with allele-specific and locus-specific oligonucleotides. The last 3′nucleotide of the allele-specific nucleotide is at the SNP. Extension past the SNP and ligation to the locus-specific oligonucleotide follow, giving rise to full-length joined products that serve as templates for PCR with universal primers and dye-labeled allele-specific primers. This method allows for high levels of multiplexing. We used oligos to analyze 1,536 heterozygous SNPs in each individual. The dye-labeled PCR products were hybridized to the genotyping array matrix and the fluorescence signals were read by the BeadArray Reader and analyzed by Genome Studio software for semi-automated genotype clustering and calling.

SNP markers were assembled into linkage groups using JoinMap 4.0 software [69]–[70]. The marker orders were obtained by maximum likelihood analysis. Linkage distances between markers were estimated using the Kosambi mapping function. The time in seconds that it took for a bee to respond with grooming motions to a Varroa mite was log transformed to approximate a normal distribution. Interval mapping was performed with MapQTL 5.0 software [71]. Chromosome-wide and experiment-wide permutation tests in which phenotypes were randomly assigned to individuals were performed to calculate significance thresholds to identify significant and suggestive QTL. The 1.5 LOD support intervals, which approximately correspond to the 95% confidence intervals for the QTL position were determined from the interval mapping LOD values and the linkage map [72] and candidate genes in this genomic region were identified.

### Analysis of Candidate Genes

The genes within the 1.5-LOD support interval were identified using the genome browser in BeeBase (http://hymenopteragenome.org/beebase/). The latest genome assembly scaffolds (Amel 4.5) within the 1.5-LOD interval were searched for annotated genes. Many of the genes are based on automated annotation software but considerable RNA sequence data has aided in these annotations. Blastp searches of the non-redundant protein databases provided a list of homologs and functional domains within the genes. Genes within the region were also assessed for putative function by reviewing the scientific literature concerning their homologs/orthologs.

## Supporting Information

Table S1 **Sequence of probes in** **Figure 1** **.**
(DOCX)

## References

[1] Sanford MT (2001) Introduction, spread and economic impact of Varroa mites in North America. In: Webster TC, Delaplane KS (Eds.), Mites of the Honey Bee. Dadant & Sons, Mansfield OH, 149–162.

[2] Genersch E (2010) Honey bee pathology: current threats to honey bees and beekeeping. Appl Microbiol Biotechnol 87: 87–97. DOI 10.1007/s00253-010-2573-8. 10.1007/s00253-010-2573-8 20401479

[3] Danka RG (2001) Resistance of honey bees to tracheal mites. Mites of the Honey Bee (T.C. Webster, K.S. Delaplane, eds) Dadant & Sons, 117–129.

[4] Schäfer MO, Ritter W, Pettis J (2010) Winter losses of honeybee colonies (Hymenoptera: Apidae): The role of infestations with Aethina tumida (Coleoptera: Nitidulidae) and Varroa destructor (Parasitiformes: Varroidae). J Econ Entomol 103 10–16: 2010. 10.1603/ec09233 20214362

[5] Dainat B, Evans JD, Chen YP, Gauthier L, Neumann P (2012) Predictive markers of honey bee colony collapse. PLoS ONE 7: e32151 doi:10.1371/journal.pone.0032151 22384162 10.1371/journal.pone.0032151 PMC3285648

[6] Oldroyd BP (2007) What’s Killing American Honey Bees? PLoS Biol 5(6): e168 doi:10.1371/journal.pbio.0050168 17564497 10.1371/journal.pbio.0050168 PMC1892840

[7] Ratnieks FLW, Carreck NL (2010) Clarity on bee colony collapse? Science 327: 152–153. 20056879 10.1126/science.1185563

[8] vanEngelsdorp D, Hayes J, Underwood RM, Pettis JS (2010) A survey of honey bee colony losses in the United States, fall 2008 to spring 2009. J Apic Res 49: 7–14.

[9] vanEngelsdorp D, Caron D, Hayes J, Underwood R, Henson M (2012) A national survey of managed honey bee 2010–11 winter colony losses in the USA: results from the Bee Informed Partnership. J Apic Res 51: 115–124.

[10] Hunt GJ (1998) The war against Varroa: How are we doing? Am Bee J 138: 372–374.

[11] Finley J, Camazine S, Frazier M (1996) The epidemic of honey bee colony losses during the 1995–1996 season. Am Bee J 136: 805–808.

[12] Vandame R, Palacio MA (2010) Preserved honey bee health in Latin America: a fragile equilibrium due to low-intensity agricultrure and beekeeping? Apidologie 41: 243–255.

[13] Arechavaleta-Velasco ME, Guzmán-Novoa E (2000) Producción de Miel de Colonias de Abejas (*Apis mellifera* L.) Tratadas y no Tratadas con un Acaricida contra Varroa jacobsoni Oudemans en Valle de Bravo, Estado de México. Rev. Veterinaria Méx. 31: 381–384.

[14] Medina-Flores CA, Guzmán-Novoa E, Arechiga-Flores CF, Aguilera-Soto JI, Gutierrez-Piña FJ (2011) Effect of *Varroa destructor* infestations on honey yields of *Apis mellifera* colonies in Mexicós semiarid high plateau. Rev Mex Cienc Pecu. 2: 313–317.

[15] Currie RW, Pernal SF, Guzmán-Novoa E (2010) Honey bee colony losses in Canada. J Apic Res 49: 104–106.

[16] Dahle B (2010) The role of *Varroa destructor* for honey bee colony losses in Norway. J Apic Res 49: 124–125.

[17] Gernersch E, von der Ohe W, Kaatz H, Schroeder A, Otten C, et al. (2010) The German bee monitoring project: a long term study to understand periodically high winter losses of honey bee colonies. Apidologie DOI: 10.1051/apido/2010014.

[18] Guzmán-Novoa E, Eccles L, Calvete Y, McGowan J, Kelly PG, et al (2010) *Varroa destructor* is the main culprit for the death and reduced populations of overwintered honey bee (*Apis mellifera*) colonies in Ontario, Canada. Apidologie 41: 443–450.

[19] Le Conte Y, Ellis M, Ritter W (2010) Varroa mites and honey bee health: can Varroa explain part of the colony losses? Apidologie DOI: 10.1051/apido/2010017.

[20] Peterson M, Gray A, Teale A (2010) Colony losses in Scotland in 2004–2006 from a sample survey. J. Apic. Res. 48: 145–146.

[21] Guzmán-Novoa E, Correa-Benítez A, Espinoza-Montaño LG, Guzmán-Novoa G (2011) Colonization, impact and control of Africanized honey bees in Mexico. Veterinaria Mexico 42: 149–178.

[22] Guzmán-Novoa E, Vandame R, Arechavaleta-Velasco ME (1999) Susceptibility of European and Africanized honey bees (Apis mellifera L.) to Varroa jacobsoni Oud. in México. Apidologie 30: 173–182.

[23] Mondragon L, Spivak M, Vandame R (2005) A multifactorial study of the resistance of honeybees *Apis mellifera* to the mite *Varroa destructor* over one year in Mexico. Apidologie 36: 345–358.

[24] Mondragon L, Martin S, Vandame R (2006) Mortality of mite offspring: a major component of *Varroa destructor* resistance in a population of Africanized bees. Apidologie 37: 67–74.

[25] Arechavaleta-Velasco ME, Guzmán-Novoa E (2001) Relative effect of four characteristics that restrain the population growth of the mite *Varroa destructor* in honey bee (*Apis mellifera*) colonies. Apidologie 32: 157–174.

[26] Currie RW, Tahmasbi GH (2008) The ability of high- and low-grooming lines of honey bees to remove the parasitic mite *Varroa destructor* is affected by environmental conditions. Can J Zool 86: 1059–1067.

[27] Tahmasbi GH (2009) The effect of temperature and humidity on grooming behavior in the honeybee, *Apis mellifera* (Hym.: Apidae) colonies against the varroa mite, *Varroa destructor* (Acari: Varroidae). J Entomol Soc Iran 27: 7–23.

[28] Andino GK, Hunt GJ (2011) A scientific note on a new assay to measure honeybee mite-grooming behavior. Apidologie 42: 481–484.

[29] Moosbeckhofer R (1997) Observations on reproduction rate of *Varroa jacobsoni* and the occurrence of mutilated mites in *Apis mellifera carnica* colonies. Apidologie 28: 193–195.

[30] Peng YS, Fang Y, Xu S, Ge L (1987) The resistance mechanism of the Asian honey bee, *Apis cerana* Fabr., to an ectoparasitic mite *Varroa jacobsoni* Oudemanns. J Invert Pathol 49: 54–60.

[31] Boecking O, Spivak M (1999) Behavioral defenses of honey bees against *Varroa jacobsoni* Oud. Apidologie 30: 141–58.

[32] Guzmán-Novoa E, Emsen B, Unger P, Espinosa-Montaño LG, Petukhova T (2012) Genotypic variability and relationships between mite infestation levels, mite damage, grooming intensity, and removal of Varroa destructor mites in selected strains of worker honey bees (*Apis mellifera* L.). J Invert Pathol (In Press). 10.1016/j.jip.2012.03.020 22465569

[33] Harbo JR, Harris JW (1999a) Selecting honey bees for resistance to *Varroa jacobsoni* . Apidologie 30: 183–196.

[34] Harbo JR, Harris JW (2005) Suppressed mite reproduction explained by the behaviour of adult bees. J Apic Res 44: 21–23.

[35] Ibrahim A, Spivak M (2006) The relationship between hygienic behavior and suppression of mite reproduction as honey bee (*Apis mellifera*) mechanisms of resistance to *Varroa destructor* . Apidologie 37: 31–40.

[36] Vandame R, Morand S, Colin M-E, Belzunces LP (2002) Parasitism in the social bee *Apis mellifera*: quantifying costs and benefits of behavioral resistance to *Varroa destructor* mites. Apidologie 33: 433–445.

[37] Tsuruda JM, Harris JW, Bourgeois L, Danka RG, Hunt GJ (2012) High-resolution linkage analyses to identify genes that influence Varroa sensitive hygiene behavior in honey bees. PLoS ONE (In Press). 10.1371/journal.pone.0048276 PMC3487727 23133626

[38] Hunt GJ, Page RE Jr (1995) A linkage map of the honey bee, *Apis mellifera*, based on RAPD markers. Genetics 139: 1371–1382. 7768445 10.1093/genetics/139.3.1371 PMC1206463

[39] Solignac M, Mougel F, Vautrin D, Monnerot M, Cornuet J-M (2007) A third-generation microsatellite-based linkage map of the honey bee, *Apis mellifera*, and its comparison with the sequence-based physical map. Genome Biol 8: R66 (doi:10.1186/gb-2007-8-4-r66) 17459148 10.1186/gb-2007-8-4-r66 PMC1896015

[40] Rasmuussen A, Matsuura T, Ruano L, Yescas P, Ochoa A, et al (2001) Clinical and genetic analysis of four Mexican families with spinocerebellar ataxia type 10. Ann Neurol 50: 234–239. 11506407 10.1002/ana.1081

[41] Hagerman KA, Ruan H, Edamura KN, Matsuura T, Pearson CE, et al (2009) The ATTCT repeats of spinocerebellar ataxia type 10 display strong nucleosome assembly which is enhanced by repeat interruptions. Gene 434: 29–34. 19171184 10.1016/j.gene.2008.12.011

[42] Blackstone C, O’Kane CJ, Reid E (2011) Hereditary spastic paraplegias: membrane traffic and the motor pathway. Nat Rev Neurosci 12: 31–42. 21139634 10.1038/nrn2946 PMC5584382

[43] Biswas S, Russell RJ, Jackson CJ, Vidovic M, Ganeshina O, et al (2008) Bridging the Synaptic Gap: Neuroligins and Neurexin I in *Apis mellifera* . PLoS ONE 3(10): e3542 doi:10.1371/journal.pone.0003542 18974885 10.1371/journal.pone.0003542 PMC2570956

[44] Knight D, Xie W, Boulianne GL (2011) Neurexins and neuroligins: Recent insights from invertebrates. Mol Neurobiol 44: 426–440. 22037798 10.1007/s12035-011-8213-1 PMC3229692

[45] Ching MSL, Shen Y, Tan W-H, Jeste SS, Morrow EM, et al. (2010) Deletions of *NRXN1* (*Neurexin-1*) predispose to a wide spectrum of developmental disorders. Am J Med Genet B, DOI 10.1002/ajmg.b.31063, 937–947. 10.1002/ajmg.b.31063 PMC3001124 20468056

[46] Craig M, Kang Y (2007) Neurexin-neuroligin signaling in synapse development. Curr Opin Neurobiol 17: 43–52. 17275284 10.1016/j.conb.2007.01.011 PMC2820508

[47] McMahon SA, Diaz E (2011) Mechanisms of excitatory synapse maturation by trans-synaptic organizing complexes. Curr Opin Neurobiol 21: 221–227. 21242087 10.1016/j.conb.2010.12.005 PMC3085653

[48] Siddiqui TJ, Craig AM (2011) Synaptic organizing complexes. Curr Opin Neurobiol 21: 132–143. 20832286 10.1016/j.conb.2010.08.016 PMC3016466

[49] Tabuchi K, Südhof TC (2002) Structure and evolution of neurexin genes: Insight into the mechanism of alternative splicing. Genomics 79: 849–859. 12036300 10.1006/geno.2002.6780

[50] Ushkaryov YA, Sudhof TC (1993) Neurexin III alpha: extensive alternative splicing generates membrane-bound and soluble forms. Proc Natl Acad Sci U S A 90: 6410–6414. 8341647 10.1073/pnas.90.14.6410 PMC46941

[51] Heisenberg M (1998) What do the mushroom bodies do for insects? Learn Mem 5: 1–10. 10454369 PMC311238

[52] Szyska P, Galkin A, Menzel R (2008) Associative and non-associative plasticity in Kenyon cells of the honeybee mushroom body. Front Syst Neurosci 2: 3 doi: 10.3389/neuro.06.003.2008 18958247 10.3389/neuro.06.003.2008 PMC2526274

[53] Biswas S, Reinhard J, Oakeshott J, Russell R, Srinivasan MV, et al (2010) Sensory regulation of *Neuroligins* and *Neurexin 1* in the honeybee brain. PLoS ONE 5(2): e9133 doi:10.1371/journal.pone.0009133 20161754 10.1371/journal.pone.0009133 PMC2817746

[54] Reichelt AC, Rodgers RJ, Clapcote SJ (2011) The role of neurexins in schizophrenia and autistic spectrum disorder. doi:2011.02.024/Neuropharm. 10.1016/j.neuropharm.2011.01.024 21262241

[55] Choi Y-B, Li H-L, Kassabov SR, Jin I, Puthanveettil SV, et al (2011) Neurexin-neuroligin transynaptic interaction mediates learning-related synaptic remodeling and long-term facilitation in *Aplysia* . Neuron 70: 468–481. 21555073 10.1016/j.neuron.2011.03.020 PMC3136118

[56] Zeng X, Sun M, Liu L, Chen F, Wei L, et al (2007) *Neurexin-1* is required for synapse formation and larval associative learning in *Drosophila* . FEBS Letters 581: 2509–2516. 17498701 10.1016/j.febslet.2007.04.068

[57] Etherton MR, Blaiss CA, Powell CM, Südhof TC (2009) Mouse neurexin-1a deletion causes correlated electrophysiological and behavioral changes consistent with cognitive impairments. PNAS 106: 17998–18003. 19822762 10.1073/pnas.0910297106 PMC2764944

[58] Rueppell O, Pankiw T, Nielsen DI, Fondrk MK, Beye M, et al (2004) The genetic architectures of the behavioral ontogeny of foraging in honeybee workers. Genetics 167: 1767–1779. 15342515 10.1534/genetics.103.021949 PMC1471018

[59] Hunt GJ, Guzmán-Novoa E, Fondrk MK, Page RE Jr (1998) Quantitative trait loci for honey bee stinging behavior and body size. Genetics 148: 1203–1213. 9539435 10.1093/genetics/148.3.1203 PMC1460054

[60] Hunt GJ, Amdam GV, Schlipalius D, Emore C, Sardesai N, et al (2007) Behavioral genomics of honeybee foraging and nest defense. Naturwissenschaften 94: 247–267. 17171388 10.1007/s00114-006-0183-1 PMC1829419

[61] Behrens D, Huang Q, Gebner C, Rosenkranz P, Frey E, et al (2011) Three QTL in the honey bee *Apis mellifera* L. suppress reproduction of the parasitic mite *Varroa destructor* . Ecol Evol 1: 451–458 doi: 10.1002/ece3.17 22393513 10.1002/ece3.17 PMC3287329

[62] Moretto G, Gonçalves LS, de Jong D (1993) Heritability of Africanized and European honey bee defensive behavior against the mite *Varroa jacobsoni*. Rev. Brasil. Genet. 16: 71–77.

[63] Harbo JR, Harris JW (1999b) Heritability in honey bees (Hymenoptera: Apidae) of characteristics associated with resistance to *Varroa jacobsoni* (Mesostigmata: Varroidae). J Econ Entomol 92: 261–265.

[64] Lodensani M, Crailsheim K, Moritz RFA (2002) Effect of some characters on the population growth of mite *Varroa jacobsoni* in *Apis mellifera* L. colonies and results of a bi-directional selection. J. Appl. Entomol. 126: 130–137.

[65] Stanimirovic Z, Jevrosima S, Nevenka A (2010) Heritability of grooming behaviour in grey honey bees (*Apis mellifera carnica*). Acta Veterinaria 60: 313–323.

[66] Espinosa L, Guzman-Novoa E, Sanchez A, Leyva N, Uribe JL, et al.. (2004) Determinación de la confiabilidad de un metodo directo para diferenciar el comportamiento de acicalamiento entre abejas de diferente genotipo. Proccedings of 11°Congreso internacional de actualización apícola. 79–86. Monterry N.L. México ANMVEA.

[67] Hunt GJ (1997) Insect DNA extraction protocol. In: Berlin, Germany: Springer-Verlag Michelli MR, Bova R, editors. Fingerprint methods based on arbitrary primed PCR. 1997: 21–24.

[68] Fan J-B, Oliphant A, Shen R, Kermani BG, Garcia F, et al. (2003) Highly parallel SNP genotyping. Pp 69–78, *Cold Spring Harbor Symposia on Quantitative Biology,* Volume LXVIII. Cold Spring Harbor Laboratory Press 0–87969–709–1/04. 10.1101/sqb.2003.68.69 15338605

[69] Stam P (1993) Construction of integrated genetic linkage maps by means of a new computer package: JoinMap. The Plant J 3: 739–744.

[70] Van Ooijen JW (2006) JoinMap version 4.0, Software for the calculation of genetic linkage maps in experimental populations. Kyazma B. V., Wageningen, Netherlands.

[71] Van Ooijen JW (2004) MapQTL 5, Software for the mapping of quantitative trait loci in expermental populations. Kyazma B. V., Wageningen, Netherlands.

[72] Dupuis J, Siegmund D (1999) Statistical methods for mapping quantitative trait loci from a dense set of markers. Genetics 151: 373–386. 9872974 10.1093/genetics/151.1.373 PMC1460471

