# Supplementary material for: Fine-Scale Linkage Mapping Reveals a Small Set of Candidate Genes Influencing Honey Bee Grooming Behavior in Response to Varroa Mites
Source: PLoS One. 2012 Nov 2;7(11):e47269. doi: 10.1371/journal.pone.0047269 (PMC3487723; doi:10.1371/journal.pone.0047269)
Supplement: Table S1 — Sequence of probes in Figure 1 . (DOCX) [file pone.0047269.s001.docx]

| >5_8439 CGCAACGTCACGCAATCCCCGATAATACTAAATACCACCGGCTACTAACGCCCCGTATAC[T/C]CTCTGCTCTGTAAAGTAGCCGGTTCGCGTGTGCCCGAAAGACGGATTTACTCGGCGCGGG |
| --- |
| >5_253573 ACCAACAAACTGCTCTTCCCGGGAAAATCAAAGGAAGCAGAAAGGCTTATAAAAGGACTT[T/C]AAGATCTATCTCGCTTCACGTACCTCTAACCAGCACCAGAATTACCGAAAACAACAGTAA |
| >5_413557 TAATCCATTATTTCTTGTAAAGAAAAATTCTTTGGTTTGCAGAAGATTTTTACGCGTTTC[A/G]TTGACTTATCACCTTAACTTTTTCATTCCCACGATAATCATGCGATGATATAACGCAGTG |
| >5_1050004 ACGATAACTGTCTCTCATTCTTCGCTTCCTTCTCCAATATTTCCAAATACCATTCAATTA[A/G]CCAAATTATCTCAAATGATCGCACGCGTCAAGTATAAATTGCATCGATTTCCATCACCAC |
| >5_1256625 GCAATATTTGTCATACACTTGACATTAATTTTCAGAAGGAAGCTAGGATGTCATGTATCA[T/C]ATTGTCGCTCAAAGATATATTAGAACACTTTTGTTCACGCAGTATAAATTCTATTATTCT |
| >5_1320383 ATTCCATCCTCTGCCGATTATAATAACTGGAGGCCACAGTTGTTGTCGGCGAGGAAGAAG[A/G]CAGGCAAATACGCGGTCAGAATCAACGATTTCCACCGAGAGAAAGGAGACATCTCCGGCC |
| >5_1544300 AGTTTCATTCTACATACTATTCCCATATATTTGCCCACGAAATGTTCAACGTACTTATGA[A/G]CAGTGGTGTAAATGGAGTATTGAGCTTTCAAGAGGATTGGACAATCTCTAAAACACGGAA |
| >5_1644271 CTCACGTGGCATCGTTTTCCTTCATCCCAGCATCCTTATTCCACGAACAGAATCCTCCGC[A/G]TCCTCCTCGTCCATTCCCATTCCCGGCCGTCGTGTTTCTTCCGTCCTTCTTCCTAGCGAG |
| >5_1868365 ATACACGTACAGTTAACCATAGCAGTATGGTAGCATCGATAAACTAACAACACACGGCAC[T/C]TCTCGTTTGGTTAAACCACCATAAATCCGGGCAACTCGTCACTATCTGCTAACTGGTTTG |

**Table S1. Sequence of probes in Figure 1.**
